# Perceptual drifts of real and artificial limbs in the rubber hand illusion

**DOI:** 10.1038/srep24362

**Published:** 2016-04-22

**Authors:** Xaver Fuchs, Martin Riemer, Martin Diers, Herta Flor, Jörg Trojan

**Affiliations:** 1 Department of Cognitive and Clinical Neuroscience, Central Institute of Mental Health, Medical Faculty Mannheim, Heidelberg University, Mannheim, Germany; 2 Aging & Cognition Research Group, German Center for Neurodegenerative Diseases (DZNE), Magdeburg, Germany; 3 Department of Psychosomatic Medicine and Psychotherapy, LWL University Hospital, Ruhr-University Bochum, Bochum, Germany; 4 Department of Psychology, University of Koblenz-Landau, Landau, Germany

## Abstract

In the rubber hand illusion (RHI), transient embodiment of an artificial hand is induced. An often-used indicator for this effect is the “proprioceptive drift”, a localization bias of the real hand towards the artificial hand. This measure suggests that the real hand is attracted by the artificial hand. Principles of multisensory integration, however, rather suggest that conflicting sensory information is combined in a “compromise” fashion and that hands should rather be attracted towards each other. Here, we used a new variant of the RHI paradigm in which participants pointed at the artificial hand. Our results indicate that the perceived positions of the real and artificial hand converge towards each other: in addition to the well-known drift of the real hand towards the artificial hand, we also found an opposite drift of the artificial hand towards the real hand. Our results contradict the notion of perceptual substitution of the real hand by the artificial hand. Rather, they are in line with the view that vision and proprioception are fused into an intermediate percept. This is further evidence that the perception of our body is a flexible multisensory construction that is based on integration principles.

The perception of our own body is malleable. What we feel as belonging to our body and where we locate our body in space strongly depends on sensory input and can easily be experimentally manipulated^1^.

In the rubber hand illusion (RHI), simultaneously brush-stroking a participant’s hidden hand and a visible artificial hand induces transient embodiment of the artificial hand^2^. A commonly used implicit measure for this embodiment is the so-called proprioceptive drift: when participants indicate the position of their hidden hand, their judgment is biased towards the position of the artificial hand.

Pavani, Spence and Driver^3^ have suggested that the dominance of vision over proprioception results in a perceptual attraction of the real hand towards the seen artificial hand. However, information from different senses is rarely combined in a “winner-takes-all” manner but rather in terms of a statistically optimal compromise in which each sensory input is weighted according to its reliability^4^. In the ventriloquism effect, for example, an auditory stimulus is spatially “captured” by a visual stimulus. It has been shown, however, that this is the case only because, under normal circumstances, vision allows more reliable localization than audition. If the reliability of the visual information is experimentally decreased, the visual and the auditory stimulus mutually attract each other^5^. As predicted by statistically optimal integration, the resulting localization is a “compromise” between the discrepant information from the two senses. This mechanism underlies several crossmodal perceptual phenomena and illusions^6^,^7^,^8^. Statistically optimal integration has also been demonstrated for localizations based on vision and proprioception^9^.

In the RHI, a conflict between vision and proprioception is present, because the seen position of the artificial hand differs from the proprioceptive position of the real hand. How discrepant information derived from vision and proprioception is integrated and how this integration relates to the experience of embodiment of the artificial hand is not yet fully understood. The interpretation based on the common proprioceptive drift paradigm does not sufficiently answer these questions. If information about the position of the hand is integrated in a statistical “compromise” fashion, attraction in the RHI should not be unidirectional but, rather, the real and the artificial hand should be attracted *towards each other*.

To clarify this question, we examined if the RHI affects the localization of the *artificial* hand, i.e. if the rubber hand drifts, too.

We conducted two experiments on the influence of the RHI on pointing movements, which only differed in respect to the instructions given to the participants. One experiment (“target: real hand”) was a replication of the classical RHI paradigm using the real hand as the target for pointing. The other experiment (“target: artificial hand”) was a novel variant in which the artificial hand itself was the target.

We used the same three conditions in both the “target: real hand” and the “target: artificial hand” experiment. Each condition consisted of an induction phase, which was specific for the condition, and a subsequent pointing phase. During induction phases in the “RHI sync” condition, the participants fixated a colored dot attached to the index finger of an artificial hand and received synchronous brushstrokes to the real and the artificial hand to induce the RHI; in the “RHI async” condition, a widely used control condition in RHI studies, they also fixated the dot and received asynchronous brushstrokes; in the “no hand” baseline condition, no artificial hand was present, no brushstrokes were received and the participants only fixated a dot presented in the same position as in the other conditions.

During the pointing phases, the participants performed a pointing movement with their right index finger while keeping their eyes closed. In the “target: artificial hand” experiment, they pointed to the dot they had fixated; in the “target: real hand” experiment, they pointed to the corresponding position of this dot on their real hand (see Fig. 1).

The “no hand” condition served as a baseline, representing localizations of the targets in the absence of visual-proprioceptive conflict. The use of this baseline allowed the estimation of drift in both the “RHI sync” and “RHI async” condition and therefore separated effects related to the embodiment of the artificial hand from effects related to the exposure to an artificial hand *per se*.

In each condition, the participants performed 9 trials, each consisting of an induction and a pointing phase.

All participants performed both experiments, which were carried out in a balanced order. Half of the sample started with the “target: real hand” and the other half with the “target: artificial hand” experiment. Within each of the experiments, the order of the three conditions was randomized.

## Results

Thirty-one participants (17 female; aged 27 ± 7 years) took part in a session comprised of the two experiments: “target: real hand” and “target: artificial hand”. One participant was excluded from each experiment due to technical problems, resulting in 30 valid datasets each.

The RHI was measured using verbal self-reports and localizations by means of pointing movements. For each experiment, we analyzed both the ratings and the localization data using linear mixed models. In the mixed models, we included the factor CONDITION (“RHI sync”, “RHI async” and “no hand”) and TIME (trial 1–9). We also computed bivariate Pearson correlations between the ratings and the localization data to test the relationship between these two dependent measures. Details on the used models are reported in the methods section. The formulae of the models and the full output of the statistical results are provided in the supplementary material.

### Vividness of the rubber hand illusion

The participants verbally rated four different statements (see Table 1 in the methods section) using a numeric rating scale ranging from 0 (no agreement) to 10 (full agreement). To attain an overall score of the perceived vividness of the RHI, we computed the average of the four ratings per trial.

To test for main effects, a linear mixed model was computed for each experiment using CONDITION and TIME as fixed factors without any interaction term.

#### Differences in vividness between the conditions

The factor CONDITION showed a significant main effect for both the “target: real hand” (*F*(1, 498) = 338, *p* < 0.001) and the “target: artificial hand” experiment (*F*(1, 501) = 388, *p* < 0.001).

In both experiments, the beta parameters for the “RHI sync” conditions were positive (“target: real hand”: b = 1.78, *t*(498) = 18.39, *p* < 0.001; “target: artificial hand”: b = 2.34, *t*(501) = 19.70, *p* < 0.001), indicating higher ratings in the “RHI sync” relative to the intercept, which represented the “RHI async” condition.

#### Changes in vividness over time

We also found a significant main effect of TIME in both experiments (“target: real hand”: *F*(1, 498) = 5, *p* < 0.001; “target: artificial hand”: *F*(1, 501) = 5, *p* < 0.001).

Because TIME was modelled as an ordered factor, 8 (k − 1) orthogonal polynomial contrasts were included in the model as regressors. Statistical tests of the TIME-components revealed that, in both experiments, the linear—and no other—component was significant (“target: real hand”: b = 0.88, *t*(498) = 6.04, *p* < 0.001; “target: artificial hand”: b = 1.07, *t*(501) = 6.01, *p* < 0.001). The positive beta parameters indicated an overall positive linear increase of vividness ratings over time (for both the “RHI sync” and “RHI async”).

In an additional model, we also tested the interaction between CONDITION and TIME, which expresses if the observed increase in the ratings over time differed between the “RHI sync” and the “RHI async” condition. In the “target: real hand” experiment, we found a statistical trend for the “RHI sync” × TIME regression coefficient (b = 0.53, *t*(504) = 1.83, *p* = 0.068). In the “target: artificial hand” experiment, the interaction was significant (*F*(1, 507) = 5, *p* = 0.024). The “RHI sync” × TIME coefficient (b = 0.80, *t*(507) = 2.26, *p* = 0.024) was positive and significant, indicating a stronger linear increase over time for the “RHI sync” than for the “RHI async” condition.

**Figure 2:**
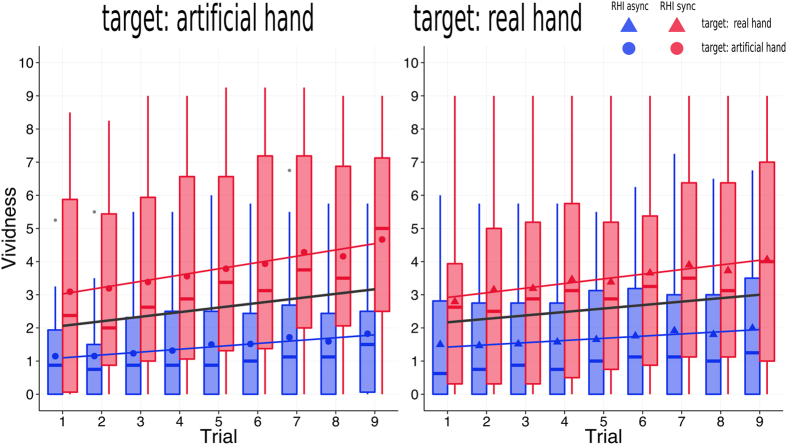
(A) Vividness ratings of the RHI in the “target: artificial hand” (left) and the “target: real hand” (right) experiment. Boxplots are shown separately for the RHI conditions (“RHI sync” as red and “RHI async” as blue) and for the 9 trials (x-axis). In addition to the median (horizontal lines in the boxplots), the mean values are highlighted as symbols (circles in the “target: artificial hand” and triangles in the “target: real hand” experiment). The whiskers extend towards the highest and the lowest observations, which are not classified as outliers. Outliers (>1.5 inter-quartile ranges above the 3^rd^ or below the 1^st^ quartile) are shown as grey circles. The solid black lines indicate the fit of an overall linear regression model (for both conditions together) relating to the significant linear effect of TIME in the linear mixed models. The colored lines indicate a linear regression fit for the two conditions separately.

The main effect of CONDITION and the linear increase of the ratings over time are shown in Fig. 2.

### Drifts in localizations

**Figure 3:**
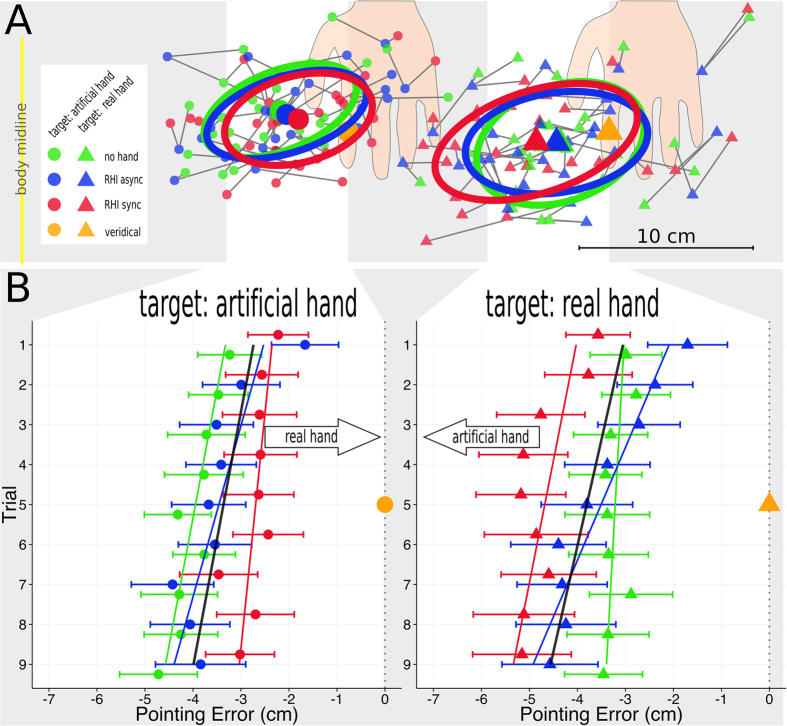
(**A**) Descriptive results from the localization tasks. The gray area of panel A corresponds to the area highlighted in Fig. 1 (annotated with the number 5) and shows aggregated localization data from both experiments in one plot. Movement endpoints were averaged over 9 trials in the three conditions. Mean values belonging to the same participant are interconnected with thin gray lines. Large symbols represent group means. Ellipses indicate 50%-borders of the estimated bivariate normal distribution. Orange symbols indicate veridical target positions. The two sections highlighted in light gray are magnified in panel B. (**B**) Pointing error along the horizontal dimension for the 9 trials separately. Note that, in accordance with panel A, pointing errors are depicted on the horizontal (x) axis and trials are represented by the vertical axis (starting with trial 1 on the top of the figure). Negative pointing error values indicate medial displacements (displacements from the target towards the body midline) and positive values lateral displacements. Symbols represent mean pointing errors; error bars represent standard errors of the mean; lines represent linear regression fit for all conditions together (black) and each condition separately (colored). Large orange symbols indicate x-positions of veridical targets.

The results of the localization task are shown in Fig. 3.

Between the conditions, we compared pointing error along the horizontal (x) dimension in which the real and the artificial hand were displaced. From all localizations, we subtracted the x-position of the veridical target to compute the pointing error values. Positive error values therefore represent an overshoot beyond the target position. Negative values represent an undershoot, indicating that the distance to the target is underestimated and that the indicated positions fall within the area between the veridical target positions and the body midline.

In the “target: real hand” experiment, negative pointing error values indicate a bias from the veridical target position towards the artificial hand. We expected a stronger bias (more strongly negative pointing error values) in the “RHI sync” condition compared to both the “RHI async” and the “no hand” condition. Since previous studies have reported a drift in localization also after asynchronous stroking^10^, we also expected a bias in the “RHI async” condition compared to the “no hand” condition. We therefore tested the following hypotheses about the pointing error values: “RHI sync” < “RHI async” < “no hand”.

In the “target: artificial hand” experiment, a bias from the veridical target position towards the real hand is represented by positive pointing error values. We expected a stronger bias (larger positive pointing error values) in the “RHI sync” condition compared to both the “RHI async” and the “no hand” condition. Again, we also expected a bias in the “RHI async” compared to the “no hand” condition. The hypotheses about the pointing error values were therefore: “RHI sync” > “RHI async” > “no hand”.

Pointing errors were fit using linear mixed models using CONDITION and TIME as fixed factors (no interaction). The hypotheses about the differences between the conditions were tested using post-hoc tests.

#### Differences in localization between the conditions

The linear mixed model indicated a significant main effect for CONDITION in both the classical “target: real hand” experiment (*F*(2, 752) = 29.8, *p* < 0.001) and the new “target: artificial hand” experiment (*F*(2, 762) = 19.6, *p* < 0.001).

In the classical “target: real hand” experiment, the results were significant for all three post-hoc comparisons between the conditions, confirming the tested hypotheses. The “RHI sync” differed significantly from the “RHI async” (difference = −1.14 cm, z = −5.70, *p* < 0.001) and the “no hand” condition (difference = −1.48 cm, z = −7.36, *p* < 0.001). The “RHI async” condition also differed significantly from the “no hand” condition (difference = −0.37 cm, z = −1.65, *p* = 0.047).

In the “target: artificial hand” experiment, we also found significant results for all three comparisons in the hypothesized direction. Again, the “RHI sync” condition differed significantly from both the “RHI async” (difference = 0.76 cm, z = 3.80, *p* < 0.001) and the “no hand” condition (difference = 1.24 cm, z = 6.21, *p* < 0.001). The “RHI async” was significantly different from the “no hand” condition (difference = 0.48 cm, z = 2.40, *p* < 0.01).

In both experiments, the linear mixed models computed a negative estimate for the model intercept, which represented the “no hand” conditions (“target: real hand”: a = −3.20 cm, *t*(30) = −4.04, *p* < 0.001; “target: artificial hand”: a = −3.94 cm, *t*(31) = −6.12, *p* < 0.001). Hence, pointing errors in the “no hand” conditions were negative, indicating a displacement from the veridical target positions towards the body midline at baseline. To test the displacements towards the body midline for the other conditions, we reformulated the linear mixed models and defined first the “RHI sync” and then the “RHI async” as the model’s intercept. In the “target: real hand” experiment, the estimates were significantly smaller than zero for both the “RHI sync” (a = −4.67 cm, *t*(30) = −5.91, *p* < 0.001) and the “RHI async” (a = −3.53 cm, *t*(30) = −4.47, *p* < 0.001) condition. This was also the case in the “target: artificial hand” experiment (“RHI sync”: a = −2.70 cm, *t*(30) = −4.19, *p* < 0.001; “RHI async”: a = −3.46 cm, *t*(30) = −5.37, *p* < 0.001). Hence, there were statistically significant displacements towards the body midline in both experiments and in all conditions.

**Figure 4:**
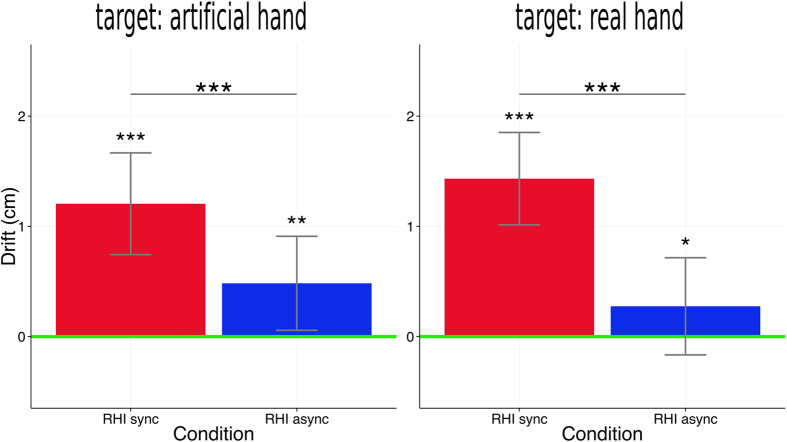
Proprioceptive drift in both experiments. Red bars indicate the mean differences between the “RHI sync” condition and the “no hand” condition and blue bars between the “RHI async” condition and the “no hand” condition. Positive values reflect drift into the hypothesized direction (towards the artificial hand in the “target: real hand” and towards the real hand in the “target: artificial hand” experiment). The means were computed by first aggregating the differences between the conditions per trial intra-individually over the 9 trials and then over the participants. The zero-line (shown in green) therefore represents the “no hand” condition. Error bars indicate standard errors of the mean (of the intra-individually aggregated values). Statistical annotations (asterisks) refer to the results of the post-hoc tests that were computed subsequent to the linear mixed models. The annotations belonging to the bars summarize p-values referring to the comparison to the “no hand” condition (*p < 0.05; **p < 0.01; ***p < 0.001).

In summary, we found a drift from the baseline (“no hand”) condition in the hypothesized direction in both experiments. The drift was significantly larger in the “RHI sync” conditions; however, there was also a drift in the “RHI async” conditions. Another result was that there were general displacements towards the body midline in all conditions of both experiments, indicating that the distances to the targets were generally underestimated (see Fig. 3A). The main results (drift values relative to the “no hand” conditions) are summarized in Fig. 4.

#### Changes in localization over time

We observed a significant main effect of TIME in both experiments (“target: real hand”: *F*(2, 752) = 5.9, *p* < 0.001; “target: artificial hand”: *F*(1, 762) = 3.9, p < 0.001).

In both experiments, the linear mixed models revealed a significant linear TIME component with a negative slope (“target: real hand”: b = −1.48, *t*(752) = −6.31, *p* < 0.001; “target: artificial hand”: b = −1.16, *t*(762) = −4.72, *p* < 0.001). In all three conditions, the values became smaller (more negative) over time, indicating increasing displacements from the targets towards the body midline. Whereas in the “target: artificial hand” experiment only the linear component was significant, there was also a significant influence of the quadratic component in the “target: real hand” experiment (b = 0.54, *t*(752) = 2.18, *p* = 0.029). The coefficient of the quadratic component, however, had a positive sign, which means that it counteracted the (negative) linear influence.

To detect differences in the influence of TIME between the conditions, we computed another model that also tested the interaction between CONDITION and TIME. In the “target: real hand” experiment, there was a significant interaction between CONDITION and the linear component. The model computed a negative interaction coefficient for the “RHI async” × linear TIME component, which was significant (b = −2.29, *t*(754) = −3.80, *p* < 0.001). Hence, the linear slope was significantly more negative for the “RHI async” than in the baseline (“no hand”) condition. There was no significant interaction between CONDITION and the quadratic TIME component. For a statistical comparison between the goodness of fit of the models with and without interaction terms, see the supplementary material.

We did not find a significant interaction between CONDITION and the linear TIME component in the “target: artificial hand” experiment, indicating that the linear slope was of comparable magnitude in all conditions.

The differences in localization between the conditions and their change over time are shown in Fig. 3B: the interaction between CONDITION and the linear TIME component in the “target: artificial hand” experiment is reflected in the different slopes for the conditions (the “RHI async” condition has a steeper slope). In the “target: artificial hand” experiment, for which no interaction was found, the regression lines run in parallel.

### Correlations between vividness and proprioceptive drift

**Figure 5:**
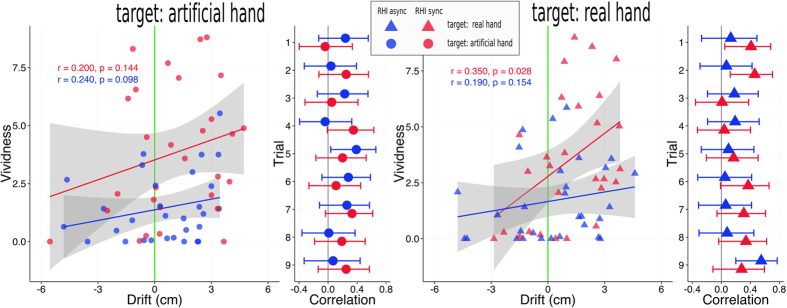
Pearson correlations between vividness and proprioceptive drift, defined as the difference between the “RHI sync” (red) and the “RHI async” (blue) to the “no hand” baseline condition, which is represented by the green zero-line. The scatter plots show correlations on the aggregated level (drift and vividness were averaged over the 9 trials within each participant). The lines and grey shaded areas represent linear regression fits (with 95% confidence intervals). The sizes of the correlation coefficients are shown in the plots together with (uncorrected) p-values for the statistical test of a positive correlation (one-sided). The circles with error bars to the right of the scatter plots show correlations between drift and vividness for each of the 9 trials separately. Error bars represent two-sided 95% confidence intervals. Note that the trials are depicted on the vertical axes.

To test if the perceived vividness of the RHI was correlated with the effects in the localization task, we computed bivariate Pearson correlations between the two dependent measures. We computed an ipsative drift measure by subtracting the baseline (“no hand”) from both the “RHI sync” and the “RHI async” condition. We coded the difference measure consistently across the experiments such that it reflected the drift from the “no hand” condition towards the respective non-target hand. To yield a stable estimate of the overall correlation of the measures, we computed correlations based on the drift and vividness scores that were averaged over the 9 trials within each participant beforehand. However, because both vividness and drift were shown to change over time (see above), we additionally computed correlations for each trial separately to test the stability of the correlations. The results are shown in Fig. 5.

In the classical “target: real hand” experiment, the correlation of the averaged scores was significant for the “RHI sync” condition (r = 0.35, *p* = 0.028). For the “RHI async” condition the correlation was positive but not statistically significant (r = 0.19, *p* = 0.154). In both conditions, the correlation coefficients were positive in 9 out of 9 independent trials, although some of the coefficients were very small. We used a binomial test to compute the likelihood of sampling 9 data sets with positive correlations from a population in which the correlation is smaller or equal to zero. The test was significant (*p* = 0.002), indicating that, in this sample, drift and vividness were most likely positively correlated for both the “RHI sync” and the “RHI async” condition.

In the novel “target: artificial hand” experiment, the correlation on the aggregated level was not significant for the “RHI sync” condition (r = 0.2, *p* = 0.144). There was a statistical trend for the “RHI async” condition (r = 0.24, *p* = 0.098). In both conditions, 8 out of 9 correlations were positive and one correlation was negative (trial 1 in the “RHI sync” and trial 4 in the “RHI async” condition). As before, we computed a binomial test, which was significant for both conditions (*p* = 0.02).

## Discussion

### Experienced vividness and localization

The rationale of the present study was to compare the classical “proprioceptive drift” measure in the RHI to a new procedure that measures localization of the artificial hand itself. We suggested that this comparison could shed light on multisensory integration processes underlying the RHI and could clarify if the perceived position of one’s real hand is *attracted by* the artificial hand or, rather, whether hand representations are *integrated* in a “compromise” fashion. We proposed that the latter mechanism likely applies to the RHI and should manifest itself in bidirectional perceptual attraction of hand representations towards an intermediate position.

In the classical “target: real hand” experiment, we replicated findings known from the literature: induction using synchronous brushstrokes (“RHI sync” condition) leads to significantly stronger personal experience (vividness) of the RHI^2^. We also replicated the “proprioceptive drift” finding, manifesting itself in localizations being displaced medially towards the artificial hand in the “RHI sync” condition, both compared to a baseline condition without visuo-tactile stimulation (“no hand”) and compared to the classical control procedure using asynchronous brushstrokes (“RHI async”). This result was very consistent as we found stronger drift in the “RHI sync” condition compared to both control conditions in all 9 trials (see Fig. 3B). It should be noted that the sizes of the effects were rather small in this study (1.48 cm compared to the “no hand” and 1.14 cm compared to the “RHI async” condition (see Fig. 4)). However, magnitudes of proprioceptive drift reported in the literature strongly vary and range from above 4 cm^11^ to below 1.5 cm^10^,^12^,^13^. Effect sizes depend on the method that is used to measure the drift, stimulation duration and the control conditions that are used. In this study, stimulation durations were relatively short (60 s in trial 1 and 30 s in the following trials). Also, drift was defined as the difference from the “no hand” condition, not in relation to a pre-test measure. This is a conservative comparison as conditions were performed in a randomized sequence and therefore temporal order effects that add to estimations of drift when compared to a pre-test were cancelled out. A comparison of subgroups from our sample supports that drift values are higher if the control condition is performed first. The average drift values were significantly higher in participants, who started with the “no hand” condition (2.52 cm; N = 9) than in participants who started with either the “RHI sync” or the “RHI async” condition (0.9 cm; N = 21), *t*(19.6) = 2.0, *p*_one-sided_ = 0.03.

In line with previous findings, the correlations between drift and vividness of the illusion were positive, albeit of small to medium size, which supports the interpretation that these two measures capture somewhat different, loosely related aspects of the RHI^14^,^15^.

In the “RHI async” control condition, we also found medial displacements compared to the “no hand” baseline, although the effect was very small. Drifts in the asynchronous condition have been reported before, for example compared to a pre-test^10^,^15^. The pre-test procedure has the limitation that localization differences can also be explained by the temporal order of conditions. As described above, the comparison with the “no hand” condition, however, should be free of potential order effects due to the randomized sequence of conditions. Therefore, our findings corroborate the claim that the “RHI async” condition has an effect on hand localization. In this respect, it is noteworthy that there was considerable variation in vividness ratings in the “RHI async” condition and that, in some participants, high vividness of the RHI was also reported during asynchronous stimulation (see Figs. 2 and 5). The correlation of drift and vividness in the “RHI async” condition was very small. However, similar to the “RHI sync” condition, it was consistent: we replicated the finding of a correlation coefficient with a positive sign in 9 out of 9 independent consecutive trials. This suggests the existence of a positive, albeit weak, relationship between vividness and drift in the “RHI async” condition. Taken together, we propose that the “RHI async” condition can also lead to a rudimentary form of embodiment of the artificial hand. These observations might contribute to a discussion, raised by Rohde *et al*.^15^, who argue that there are effects of the asynchronous condition on perceived hand location that are not sufficiently taken into account in the RHI literature.

The aim of this study was to test if there is a complementary drift from the artificial hand towards the real hand. We therefore implemented the “target: artificial hand” experiment. Due to the identical induction of the RHI, both experiments yielded comparable results in respect to the experienced vividness of the RHI. However, for the localization task, we found a pattern of results that was complementary to the one found for the classical “target: artificial hand” procedure: in the “RHI sync” condition, we found lateral displacements of the artificial hand—that is, towards the real hand—both compared to the “no hand” baseline condition and the “RHI async” control condition. Again, there was a lateral displacement in the “RHI async” condition compared to the “no hand” condition, indicating that the “RHI async” condition had an effect on localization.

It remains unclear, however, if there is a linear relationship between perceived vividness and drift in this novel paradigm, similar to the classical variant. Although correlations between drift and vividness were positive in the 8 of 9 trials of the “RHI sync” condition, the coefficients were weak and on an aggregated level, the correlation was not statistically significant. The correlations of vividness and drift in the “RHI async” condition were also small and mostly had a positive sign (in 8 of 9 trials). On the aggregated level, we found a statistical trend. It can be assumed that there were positive associations (in both conditions) in the new paradigm as well, but that their effect sizes were very small (smaller than in the classical variant). To reliably detect a very small positive correlational effect of r = 0.2, a sample size of 152 is required (at a power of 0.8 and an alpha of 0.05). Therefore, our design was underpowered to reliably test if vividness and drift correlate in the “target: artificial hand” experiment.

There were displacements towards the body midline in all conditions of both experiments, including the baseline (“no hand”) conditions. Irrespective of illusion-related effects, the distances to the veridical target positions were systematically underestimated. Underestimation of the distance to the hand at baseline has been reported before, using a similar setup and similar methods^11^. This mismatch between veridical and perceived positions of the targets needs to be taken into account in the analysis and interpretation of the data. Illusion-related effects need to be interpreted as differences between the perceived positions in different experimental conditions and not with respect to veridical target positions.

For this reason, the drift in the “RHI sync” in the “target: artificial hand” experiment is a drift away from the perceived position of the artificial hand at baseline (represented by the “no hand” condition). For the interpretation of this effect, it is irrelevant that the resulting location in the “RHI sync” condition is closer to the veridical position of the artificial hand (see Fig. 3A).

Taken together, our new variant of the RHI reveals that the drift observed in RHI experiments depends on the target: when the participants point at the real hand, they are biased towards the artificial hand and when they point at the artificial hand, they are biased towards the real hand. Apparently, spatial representations of the real and artificial hand converge towards each other.

We suggest that this convergence of localizations may be explained by a process in which the conflicting representation of hand positions derived from vision (artificial hand) and proprioception (real hand) are combined into an intermediate hand percept, according to principles of multisensory integration described by Ernst and Banks^4^. This integrated “phantom” representation might then influence both the localization of the real hand and of the artificial hand. According to the theoretical framework by Ernst and Banks^4^, it could be expected that participants point to this intermediate “phantom” in both experiments. It is noteworthy, however, that the position judgments from both experiments did not fully converge, that is, the participants did not point to the same intermediate location in both experiments. Rather, there was an obvious gap between the localizations (see Fig. 3A). There are, however, notable differences between the RHI paradigm and experiments that were designed to investigate principles of multisensory integration according to the framework proposed by Ernst and Banks^4^ which might explain why the participants did not point to the assumed intermediate percept directly. In studies that were designed to test the theoretical framework by Ernst and Banks^4^,^5^, conflict between sensory modalities was manipulated in a subtle way, not consciously perceived by the participants. In the RHI, however, the relatively large spatial discrepancy between the real and the artificial hand positions is quite obvious to the participants. Since the participants are aware of this discrepancy and their instruction clearly consists in localizing either the real hand or the artificial hand (and not an “intermediate percept”), it is possible that the participants made use of their prior knowledge about the veridical hand positions when they performed the localization task. This might give rise to a compensation mechanism that prevents them from pointing toward the integrated percept directly. The observed shifts in the localization data might therefore represent an implicit spatial attraction towards the integrated percept rather than the location of the “phantom” itself.

However, there are also other theoretical explanations for these results. Possibly, multiple spatial representations of the hands, relating to the visual cue, the proprioceptive cue, and a combination of both, exist simultaneously and are accessible when participants perform the localization task. This type of integration is referred to as non-mandatory fusion and has for example been shown to apply in the integration of visual and haptic information^16^. There might be variation between participants or even within the same participant with respect to the spatial information that is used in the pointing task. This could result in smaller displacements in the localizations, as reported in this study. Another possibility is that the integration in the RHI is inherently unstable due to the large spatial conflict between the visual and the proprioceptive representation of the hands. There are several Bayesian accounts that provide a theoretical framework for a transition between integration of cues and breakdown of integration^17^, when discrepancies are large. A Bayesian approach to robust cue integration^18^ assumes that priors are composed of a complex mix of distributions and that participants decrease the weighting of cues when conflict increases but, even at larger conflicts, do not fully veto them. This model provides good fit to participants’ performance in perceptual tasks with large conflicts. Causal inference models^19^ approach perception in a fashion that is similar to a decision process in which is inferred if cues stem from the same or from different origins. These models could both allow the possibility that, given that the sensory conflict in the RHI is large, states of integration or segregation into two separate percepts (a real hand on the table and an (unrelated) artificial hand) might become unstable. Possibly, this could give rise to perception switching between a state in which the hands are perceptually integrated and a state in which their integration breaks down. The result of such switching of states that might occur between (or even within) single trials which could result in the participants sometimes attempting to point at an integrated percept and sometimes ignoring one modality and pointing to a percept that is only based on proprioception or only on vision. On average, this could result in small convergences as seen in our data. The idea that the RHI becomes unstable with larger discrepancies between the real hand and the artificial hand is also in line with the fact that the vividness of the RHI decreases with increasing distance between the hands^20^. However, the effect of increasing spatial discrepancy of the hands on proprioceptive drift has, to our knowledge, not systematically been investigated.

Finally, sensory recalibration (or remapping) is yet another potential mechanism that could explain our results. Recalibration of sensory cues occurs when a systematic conflict between senses remains stable over some time and cues are available that allow to infer which cue is biased. Estimation of such a bias and recalibration can be integrated into the above-mentioned Bayesian models^17^,^18^. A typical example is prismatic adaptation. In the RHI, observing touch on the seen hand that is highly temporally correlated to the sensation of touch in a different location provides feedback that some cue might be erroneous and give rise to recalibration of the cue. Our data therefore suggests that not only one cue but that, rather, both vision and proprioception are recalibrated. Recalibration of proprioception is a process that has been proposed in theoretical accounts on the RHI^21^,^22^. However, these accounts provide an explanation for recalibration of proprioception but do not take into account that vision might also be object of recalibration.

Our data does not allow determining the precise mechanism that underlies the integration process. This would require a setup in which sensory information about hand positions were manipulated subtly or in which prior information about hand positions can be manipulated. This kind of modeling is not possible using the standard RHI paradigm. Though the exact mechanism remains unclear, our data show that integration—not substitution—principles are involved in the RHI.

### Effects of time

Our experiments also revealed that the factor TIME (from trial 1 to trial 9) played a significant role both in the personal experience of the RHI and in the localization task. In both experiments, the vividness ratings of the illusion in both the “RHI sync” and “RHI async” linearly increased, suggesting that the illusion became stronger with every repetition of the induction. In both experiments, the slope was slightly steeper for the “RHI sync” condition, indicating that the linear increases were somewhat stronger for the “RHI sync” conditions.

The localizations also changed over time. In the classical “target: real hand” experiment, negative pointing errors (from the target positions towards the body midline) became stronger suggesting that, over time, the participants more pronouncedly undershot, i.e. underestimated the distance to the targets. Additionally, there were differences in the slope between the conditions: while undershoot moderately increased in the “RHI sync” and the “no hand” condition, the effect was significantly stronger in the “RHI async” condition (see Fig. 3B). Over time, the differences between the “RHI async” and the “RHI sync” that were clearly visible in the first trials decreased and almost disappeared in the last trials. This is in accordance with results by Rohde *et al*.^15^ who also observed that proprioceptive drift in the asynchronous control condition increased over time, if judgments were performed repeatedly. Another result that relates to the stronger effect in the “RHI async” condition is that in the first trials there was a negative drift for in the “RHI async” condition. In these trials, participants pointed further laterally (contrary to the hypothesized direction) in the “RHI async” than in the “no hand” condition (see Fig. 3B). Negative drifts in the asynchronous condition (compared to pre-test measures) have also been reported before^10^,^12^,^13^,^23^,^24^.

In the new “target: artificial hand” procedure we observed an increase in undershoot as well. In contrast to the classical experiment, this increase was comparable for all conditions, including the “no hand” baseline condition. This result is different from what could have been expected given our theoretical assumption of merging representations of the hands in the “RHI sync” condition. In the “target: artificial hand” experiment, the direction of the bias towards the body midline is opposite to the embodiment-related drifts. Since the vividness of the RHI became stronger over time, it could have been assumed that the “RHI sync” condition would not (or to a lesser degree) be subject to increasing bias towards the body midline.

The reasons why participants increasingly undershot in both the classical and the novel paradigm are not quite clear but it might play a role that pointing was generally biased towards the body midline, even at baseline. In case of the “target: real hand” experiment, it is possible to argue that this tendency increased over time because—independently of visuo-tactile stimulation—the perceived position of the hidden hand drifted towards the body midline, a phenomenon reported before^25^,^26^. However, this cannot explain why baseline bias also increased over time in the “target: artificial hand” experiment, in which the target was not proprioceptive. This effect could rather reflect shifts of the frame of reference that is used when performing hand movements with closed eyes. It remains unclear why this tendency was especially pronounced in the “RHI async” condition of the classical “target: real hand” experiment and why there was no such interaction in the “target: artificial hand” experiment. It is possible, however, that these differences between the experiments are related to asymmetries of the experimental setup. In the classical “target: real hand” experiment, time-related changes in localization and embodiment-related drifts add up whereas in the new “target: artificial hand” experiment they should rather counteract each other. Secondly, the distance to the targets in the “target: real hand” was greater (60 cm vs. 45 cm from the middle starting position) and a different type of arm-movement was necessary to perform the task. This might give rise to different drifts and dynamics in the drifts over time, as efforts of movements were probably higher in the “target: real hand” experiment.

In summary, the effects of time suggest that there are dynamic mechanisms involved in the RHI that are insufficiently understood. Changes in the localization judgments over time might either reflect (1) slow changes in proprioceptive awareness that results from sensory deprivation from the immobilized arm or (2) shifts of the spatial frame of reference. The increase over time in the vividness of the illusion could reflect a mechanism of learning or long-term plasticity of the body image related to extended exposure to the RHI.

## Conclusion

We found convergence of localization of the real hand and the artificial hand in the RHI. We propose that in the RHI, spatially discrepant representations of the hand position derived from proprioception (real hand) and vision (artificial hand) are integrated into an intermediate “phantom” representation. Integration into such a “compromise” could be explained by processes of multisensory integration described by Ernst and Banks^4^. While the data do not support the interpretation that localizations reflect the position of this assumed “phantom” directly, it is possible that there is an implicit perceptual attraction towards this combined representation. However, other explanations assuming that multiple representation exist simultaneously, that integration is unstable or that sensory cues are recalibrated can also explain the spatial convergence we observed. In either way, this study demonstrates that integration—not substitution—processes predominate in the RHI. Further studies (using different experimental setups) are necessary to clarify the underlying mechanisms.

Our results also suggest that common interpretations of the RHI need to be reexamined: the often-reported drift that is usually interpreted as a drift towards *the artificial hand* might rather be a drift towards a combined percept that neither spatially matches the real nor the artificial hand representation. This idea contradicts the notion of perceptual substitution of the real hand by the artificial hand^27^. Rather, percepts of the real and artificial hand are—in a very literal sense—“confused” with each other.

## Methods

### Participants

In total, 31 participants were included in the analyses; of them 17 were female and 22 students. All participants were right-handed. Their age ranged between 21 and 51 years (m = 26.9; sd = 6.9). From each of the experiments, one participant was excluded due to technical problems. Details on power analyses and criteria for selection and exclusion are provided in the supplementary material 1. The study was approved by the Medical Ethics Commission II of the Medical Faculty Mannheim, Heidelberg University and all methods were carried out in accordance with the ethical guidelines. All participants provided written informed consent according to the institutional guidelines.

### Induction of the rubber hand illusion (RHI)

Induction of the RHI was achieved by stroking the real and the artificial hand using soft paint brushes (“CLICK & GO”, Faber Castell, Stein, Germany). The artificial hand was a life-sized and sex-matched prosthetic glove with a naturalistic shape, color and texture (Otto Bock, Duderstadt, Germany). Synchronous brush-stroking of congruent parts of the real and the artificial hand is known to induce the RHI; asynchronous stroking is commonly used as a control procedure^2^. We applied brushstrokes to the dorsum of the third digit in a proximal-distal direction, starting at the proximal phalanx and ending at the distal phalanx just before touching the fingernail. During stimulation phases, participants fixated a colored dot attached to the medial interphalangeal joint of the artificial hand’s index finger. Hence, this reference area was close to (2.5 cm distance) but not identical to the brushed area. The reason for the use of two different areas was that brush-stroking the reference area itself would have interrupted participants’ sight. Due to the proximity of the reference to the brushed area, the brush strokes were in the participants’ focus of attention. The distance between the index fingers of the real hand and the artificial hand was 15 cm. In order to standardize the number and frequency of brushstrokes between conditions and participants, we used audio tracks, played to the experimenter via stereo headphones during stimulation phases. Beeps presented on the left channel signaled brushstrokes to the artificial hand (performed with the experimenter’s left hand) and beeps on the right channel signaled strokes to the participant’s real hand (performed with the experimenter’s right hand). Beeps were presented at a mean frequency of 0.5 Hz. In order to render the stroking pattern less predictable, we jittered the onsets of the beeps by adding Gaussian temporal noise with a mean of 0 s and a standard deviation of 0.2 s. For the “RHI async” condition, two independent channels were created and one of them was shifted by half of a period (1 s). To ensure a fully developed RHI, the stimulation durations were 60 s for the first trial of one condition block. The stimulation in the subsequent 8 trials lasted for 30 s.

### Ratings of the vividness of the RHI

Following each trial of the “RHI sync” and “RHI async” conditions, we asked the participants to rate their agreement with four statements. The ratings were given verbally using a numeric rating scale ranging from 0 (no agreement) to 10 (complete agreement). The statements were adapted from Longo *et al*.^28^ and have been shown to capture differential aspects of experiencing the RHI. The statements are shown in Table 1.

### Performance of pointing movements

Pointing movements were performed with closed eyes, using the index finger of the right hand. During pointing, the head and the eyes were kept still, directed towards the opposite wall. The participants were instructed to perform pointing movements in a natural and fluent way, at once, without interruptions. They carried out practice trials beforehand to become acquainted with the procedure.

Each of the conditions consisted of 9 trials including induction phases, pointing phases and (in the case of “RHI sync” and “RHI async” conditions) ratings.

The principle course of a trial was identical in all conditions of both experiments. We used 9 different starting positions in randomized order to avoid monotonous, stereotypical motor behavior and learning during the course of the experiment. Depending on the starting position that was used, the distance (in the horizontal direction) from the starting positions was between 40 and 50 cm in the “target: artificial hand” and between 55 and 65 cm in the “target: real hand” experiment.

The exact course of one trial was as follows: the participants (1) were told which of the 9 starting positions to use and positioned their right index finger there; (2) fixated a cross on the opposite wall for 5 s which made sure that the participants did not see the visual reference and/or the artificial hand before the beginning of the induction phases; (3) directed their gaze to a visual reference (a colored dot) that was—depending on the condition—either attached to the artificial hand or a thin metal rod, resulting in an identical spatial position; (4) observed the visual reference while (in “RHI sync” and “RHI async” conditions) their hidden real hand and the artificial hand was touched with paint brushes; (5) fixated the cross again and immediately closed the eyes before the setup was transformed by turning over the cover board; (6) performed a pointing movement towards the target and moved the hand back to the starting position with closed eyes; (7) opened the eyes again after the setup had been changed back to its original form by putting the board upright again; (8) answered four questions on the experienced vividness of the illusion (in “RHI sync” and “RHI async” conditions only).

During pointing, the participants had no visual feedback about the target position and their accuracy, because their eyes were closed throughout the movement and were not opened before the right hand had been returned to the starting position. There was also no haptic feedback, since the fingertip only touched the wooden board mounted above the real and artificial hand and never the target positions or other objects that might have given information about task performance.

### Recording of pointing movements

We used a Polhemus Patriot 6 DOF electromagnetic motion tracking system (Polhemus, Colchester, VT, USA) for capturing the pointing movements. This system measures the position of a sensor relative to a source with high spatial and temporal accuracy. The Patriot’s especially small and light “Teardrop” sensor was attached to the fingernail of the participant’s right index finger with adhesive tape. The patriot system was connected to a Windows PC via the RS-232 serial bus. Data recording and timing of procedures was controlled with self-programmed software written in the Python programming language (www.python.org). Movement trajectories were captured continuously at a sampling rate of 60 Hz.

### Analysis of pointing movements

Initially, we removed data containing trajectories that had been classified as missing or invalid during testing (e.g., if participants did not follow the instructions or if technical problems occurred). As described in the supplementary material, this led to the exclusion from analysis of one participant in each experiment because >33% of the data was missing due to technical problems during testing. This resulted in 30 complete cases per experiment. In the remaining cases, 15 single trials were removed in total from both experiments, overall affecting less than 1% of the data.

We then performed quality control of the measurement accuracy of the Polhemus Patriot motion tracking system. We did so by comparing theoretical coordinates to coordinates captured during a calibration procedure, which was executed before each experiment. The calibration data also allowed co-registration of localization data across participants. For group analysis, we transformed individual data into a common space by applying a linear transformation to the data. For this, we used the freely available “absor” script (http://www.mathworks.com/matlabcentral/fileexchange/26186-absolute-orientation-horns-method) for Matlab (MathWorks Inc., Natick, MA, USA). In each individual case, this procedure led to satisfying registration. We found no indication of nonlinear distortions within the measured area, which can arise from distortions in the magnetic field by metal objects.

We extracted trajectory endpoints using a criterion that determined the coordinates of the participants’ first contact with the cover board. Trajectories and extracted endpoints were visually inspected for plausibility using the “rgl” package^29^ for the R programming language (version 3.1.0)^30^. We removed 11 trajectories (less than 1% of the data) in which the applied algorithm did not yield a valid endpoint of the trajectory.

### Statistical Analyses

All statistical analyses were performed using R^30^. We analyzed pointing data and subjective ratings with linear mixed models using the “lme4” package^31^. P-values for the statistical tests of the model parameters were determined using the “lmerTest” package^32^. Full model formulation and output is provided in the supplementary material. Graphics were created using the “ggplot2” package^33^ for R.

#### Statistical models for analyzing the vividness of the RHI

Statistical analyses of the vividness scores were performed using a linear mixed model for each experiment. We used CONDITION as a fixed within-subject factor with two levels (“RHI async” and “RHI sync”), TIME as an ordered fixed within-subject factor with 9 levels (for the 9 trials) and PARTICIPANT as a random-intercept factor. Within the factor CONDITION, we defined the “RHI async” condition as the baseline level. As a consequence, the linear mixed model maps the “RHI async” condition onto the intercept and the deviations from the intercept of the other conditions (here only the “RHI sync” condition) are expressed as beta coefficients. The “no hand” condition was not included in these models, because no ratings were given in this condition. Because the factor TIME (trial 1–9) had a temporal order, we used orthogonal polynomial contrasts to model the time effects. In total, the model included 8 (k −1) components that were used as regressors (linear, quadratic, cubic et cetera).

We first tested the influence of CONDITION and TIME by including them into the model in an additive fashion (without an interaction term). In a second model, we tested the interaction between CONDITION and TIME. For simplicity of model comparisons, we tested the linear component of TIME (the only significant component) directly, by including it into the model as a numeric regressor. We omitted the 7 remaining, non-significant orthogonal polynomial contrasts.

#### Statistical models for the localization data

We analyzed differences in localization along the horizontal (x) dimension in which the real and the artificial hands were displaced and ignored the y-dimension in which they were aligned. From all localizations, we subtracted the x-position of the veridical target to compute the pointing error.

Statistical analyses were performed based on the pointing error values. We computed a linear mixed model for each of the two experiments. We used CONDITION as a fixed within-subject factor with three levels (“no hand”, “RHI async”, “RHI sync”), TIME as an ordered within-subject factor with 9 levels (using orthogonal polynomial contrasts), and PARTICIPANT as a random-intercept factor. Within the factor CONDITION, we defined the “no hand” condition as the baseline level. Hence, this condition is represented by the model’s intercept.

To test the hypotheses about the differences in pointing error values between the conditions (“proprioceptive drift”), we used post-hoc tests. Post-hoc tests and p-value adjustments were carried out using the “multcomp” package^34^ for R. P-values were adjusted for multiple testing using the false discovery rate (FDR).

To test for interactions between CONDITION and TIME, we used the same approach as for the vividness scores. For each experiment, we computed another model, which included interactions between CONDITION and the significant TIME components (in the “target: real hand” experiment the linear and the quadratic component; in the “target: artificial hand” experiment only the linear component) and omitted the remaining, non-significant components.

## Limitations and Future Directions

The present study showed that there is a complementary drift from the artificial hand towards the real hand, which is in line with concepts of statistically optimal integration. However, as discussed above, there was a gap between the localizations from both experiments, which leaves open questions on whether the representations are combined into one representation and, if so, why the participants did not point at the integrated percept. We propose that either prior knowledge about hand positions had an effect on localization or that integration was unstable leading to participants pointing at different representations in different trials. These questions cannot be answered by our data. To closer examine the principles of multisensory integration, it would be necessary to study localization under conditions in which the conflict between seen position of (artificial) hands and perceived hand position (proprioception) can be manipulated in a more subtle way so that participants are not consciously aware of the conflict. This could reduce the potential role of prior knowledge and of breakdown of integration due to large discrepancies between the senses. The RHI paradigm is not suitable for this type of manipulation. It might, however, be possible to realize such manipulation of visual input using prism displacements or virtual or augmented reality. Proprioception could be manipulated using passive manipulation of arm position or tendon vibrators.

Furthermore, to test if integration of vision and proprioception is indeed statistically optimal, the mode of judgment needs to be taken into account as well. In our experiment, the mode was proprioceptive because the participants performed hand movements with closed eyes. To determine statistical optimality, single cue conditions (vision-only and proprioception-only) would be necessary. Such modeling to determine contributions of vision and proprioception to perceived limb position is challenging but possible^35^.

## Additional Information

**How to cite this article**: Fuchs, X. *et al*. Perceptual drifts of real and artificial limbs in the rubber hand illusion. *Sci. Rep.* **6**, 24362; doi: 10.1038/srep24362 (2016).

## Supplementary Material

Supplementary Information

## Figures and Tables

**Figure 1:**
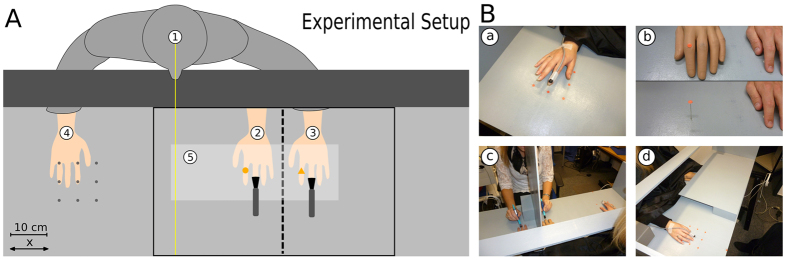
(**A**) Experimental setup. In all conditions, the participants (1) sat at a table in a standardized position (the body midline is shown as a yellow line) and completed 9 consecutive trials, each comprised of an induction and a pointing phase. In induction phases, the participants observed a visual reference (here shown as an orange circle) presented in the same position for all conditions. This reference consisted of a colored dot either attached to the index finger of a visible artificial hand (in “RHI sync” and “RHI async” conditions) (2) or to a thin metal rod (in “no hand” conditions). The RHI was elicited by tactile stimuli applied at the dorsum of the third digit using two soft brushes. The left hand (3) was occluded from sight by a vertical screen (dashed line). In pointing phases, the screen was turned over, covering the area indicated by solid black lines. Pointing movements were performed with the right hand (4) and closed eyes, starting at one of 9 starting positions (grey points) in randomized order. In the “RHI sync” and “RHI async” conditions, the participants rated the personal experience of the RHI using standard questions (see Table 1 in the methods section) at the end of each trial. The two experiments only differed with respect to the instructed target position: in the “target: artificial hand” experiment, the target position was identical to the reference position (orange circle); in the “target: real hand” experiment, the participants pointed at the corresponding position on the index finger of their real left hand (orange triangle); (**B**) Photos of the experimental setup. (a) A position sensor was attached to the index finger of the participants’ right hand to record the movements. (b) A visual reference (a colored dot) was either attached to an artificial hand (upper picture) or mounted on a thin metal rod with a height of 4.5 cm (lower picture), which allowed its presentation in the same location as in “RHI sync” and “RHI async” conditions (where it was attached to the artificial hand). (c) During the induction phases, a vertical board occluded the real hand from sight. (d) During the pointing phases, the same board was turned over, covering the area relevant for pointing movements.

**Table 1:** Statements of perceived vividness of the RHI.

**No.**	**Statement**
1	“It seemed like I was feeling the touch of the paintbrush in the location where I saw the rubber hand being touched”.
2	“It seemed like the rubber hand was my hand”.
3	“It seemed like my hand was in the location where the rubber hand was”.
4	“It seemed like I couldn’t really tell where my hand was”.
