# Supplementary material for: Perceptual drifts of real and artificial limbs in the rubber hand illusion
Source: Sci Rep. 2016 Apr 22;6:24362. doi: 10.1038/srep24362 (PMC4840308; doi:10.1038/srep24362)
Supplement: Supplementary Information [file srep24362-s1.pdf]

‘Perceptual drifts of real and artificial limbs in the rubber hand illusion’: Supplementary Material

Xaver Fuchs, Martin Riemer, Martin Diers, Herta Flor, and Jörg Trojan

Supplementary material part 1: additional information on a-priori power analyses, stopping rules and exclusion of participants

Estimation of sample size and stopping rules

Before the experiment, we conducted a-priori power analyses to estimate adequate sample sizes. Expected effect sizes were based on results reported by Longo et al. (2008). In our study, four questions from Longo et al. (2008) were used to measure the vividness of the rubber hand illusion. From means and standard deviations provided in the online supplementary material belonging to the study by Longo et al. (2008), we calculated Cohen’s d by dividing the difference of the means (synchronous minus asynchronous condition) by the pooled standard deviation (see Cumming, 2012, p291). The average effect size of the four questions was 0.61. We used the package “pwr” (Champely, 2012) for the statistical software R (R Core Team, 2014) to perform power analyses. For the use of a one-tailed paired t-test design, power analysis using an alpha parameter of 0.05 and a power of 0.95 indicated a minimum sample size of 30 and of 18 if a power of 0.8 was desired.

We also conducted a power analysis based on the proprioceptive drift reported in Longo et al. (2008). Compared to a pre-test, the authors find a significant drift of 1.34 cm for the synchronous condition ($t(119) = 4.56$, $p < 0.001$). This corresponds to an effect size of $d = 0.59$, comparable to the ones found for the self-reports (see above). Power analyses indicated a minimum sample size of 32 if a power of 0.95 and of 19 if a power of 0.8 was desired.

We therefore concluded that a sample size of 30 might be sufficient to reveal the assumed effects.

Hence, we used the criteria of 30 complete datasets as a stopping rule for data acquisition.

Exclusion of participants

Due to problems during testing, we lost data. One participant was immediately removed from both experiments due to overtly uncooperative behavior during testing. Two other participants were removed from only one of the experiments (one from the “target: artificial hand” and one from the “target: real hand” experiment) due to > 33% missing data caused by technical problems during testing. Accordingly, the total number of invited participants was 32 resulting in 30 complete datasets in both experiments.

References

Champely, Stephane (2012). pwr: Basic functions for power analysis. R package version 1.1.1. http://CRAN.R-project.org/package=pwr.

Cumming, G. (2012). Understanding the New Statistics: Effect Sizes, Confidence Intervals, and Meta-Analysis. Routledge, Taylor & Francis Group.

Longo, M. R., Schüür, F., Kammers, M. P. M., Tsakiris, M., & Haggard, P. (2008). What is embodiment? A psychometric approach. *Cognition, 107*(3), 978–998.

R Core Team (2014). R: A language and environment for statistical computing. R Foundation for Statistical Computing, Vienna, Austria. URL http://www.R-project.org/.

Supplementary material part 2: formulation and results of linear mixed models

Contents

1. Models for RHI vividness **1**

1.1. "Target: real hand" experiment 1

1.1.1.: Model without interaction between CONDITION and TIME 1

1.1.2.: Model with an interaction term between CONDITION and TIME (linear component) 3

1.1.3.: Comparison between the model without an interaction term and model with interaction using likelihood ratio test 4

1.2. "Target: artificial hand" experiment 4

1.2.1.: Model without interaction between CONDITION and TIME 4

1.2.2.: Model with an interaction term between CONDITION and TIME (linear component) 5

1.2.3.: Comparison between the model without an interaction term and model with interaction using likelihood ratio test 6

2. Models for localization data **6**

2.1. "Target: real hand" experiment 6

2.1.1.: Model without interaction between CONDITION and TIME 6

2.1.2.: Model with an interaction term between CONDITION and TIME (linear and quadratic component) 8

2.1.3.: Comparison between the model without an interaction term and model with interaction using likelihood ratio test 9

2.1. "Target: artificial hand" experiment 10

2.2.1.: Model without interaction between CONDITION and TIME 10

2.2.2.: Model with an interaction term between CONDITION and TIME (linear component) 11

2.1.3.: Comparison between the model without an interaction term and model with interaction using likelihood ratio test 12

1. Models for RHI vividness

1.1. "Target: real hand" experiment

1.1.1.: Model without interaction between CONDITION and TIME

1.1.1.1.: Anova table

```
## Analysis of Variance Table of type III  with  Satterthwaite
## approximation for degrees of freedom
##           Sum Sq Mean Sq NumDF DenDF F.value  Pr(>F)
## Condition 424.70  424.70     1   498  338.17 < 2e-16 ***
## Trial      48.08    6.01     8   498    4.79 1.1e-05 ***
## ---
## Signif. codes:  0 '***' 0.001 '**' 0.01 '*' 0.05 '.' 0.1 ' ' 1
```

1.1.1.2.: Model summary table

```
## Linear mixed model fit by REML t-tests use Satterthwaite approximations
##   to degrees of freedom [lmerMod]
## Formula: Vividness ~ Condition + Trial + (1 | Participant)
##    Data: Data.TargetRealHand.Vividness
##
## REML criterion at convergence: 1783
##
## Scaled residuals:
##     Min      1Q  Median      3Q     Max
## -4.4828 -0.6461 -0.0583  0.6381  5.1057
##
## Random effects:
##  Groups      Name        Variance Std.Dev.
##  Participant (Intercept) 4.882    2.210
##  Residual                1.256    1.121
## Number of obs: 537, groups:  Participant, 30
##
## Fixed effects:
##                    Estimate Std. Error        df t value Pr(>|t|)
## (Intercept)         1.71259    0.40919  29.80000   4.185 0.000231 ***
## ConditionRHI sync   1.77916    0.09675 498.00000  18.389  < 2e-16 ***
## Trial.L             0.87978    0.14560 498.00000   6.042 2.98e-09 ***
## Trial.Q             0.05022    0.14545 498.00000   0.345 0.730048
## Trial.C             0.04424    0.14525 498.00000   0.305 0.760793
## Trial^4             0.01582    0.14520 498.00000   0.109 0.913295
## Trial^5             0.10503    0.14509 498.00000   0.724 0.469476
## Trial^6             0.10341    0.14488 498.00000   0.714 0.475718
## Trial^7             0.10207    0.14473 498.00000   0.705 0.480994
## Trial^8            -0.04263    0.14468 498.00000  -0.295 0.768374
## ---
## Signif. codes:  0 '***' 0.001 '**' 0.01 '*' 0.05 '.' 0.1 ' ' 1
##
## Correlation of Fixed Effects:
##             (Intr) CnRHIs Tril.L Tril.Q Tril.C Tril^4 Tril^5 Tril^6 Tril^7
## CndtnRHIsyn -0.118
## Trial.L      0.001 -0.003
## Trial.Q      0.001 -0.001  0.011
## Trial.C      0.000  0.001  0.007  0.008
## Trial^4      0.000  0.003  0.002  0.005  0.007
## Trial^5      0.000  0.003  0.000  0.002  0.005  0.006
## Trial^6      0.000  0.002 -0.001  0.001  0.003  0.004  0.004
## Trial^7      0.000  0.001 -0.001  0.000  0.001  0.002  0.002  0.001
## Trial^8      0.000  0.000  0.000  0.000  0.000  0.001  0.001  0.001  0.000
```

1.1.2.: Model with an interaction term between CONDITION and TIME (linear component)

1.1.2.1.: Anova table

```
## Analysis of Variance Table of type III  with  Satterthwaite
## approximation for degrees of freedom
##                    Sum Sq Mean Sq NumDF  DenDF F.value    Pr(>F)
## Condition          425.18  425.18     1 504.00  343.64 < 2.2e-16 ***
## Trial.L             45.71   45.71     1 504.01   36.94 2.404e-09 ***
## Condition:Trial.L   4.14    4.14     1 504.01    3.35   0.06781 .
## ---
## Signif. codes:  0 '***' 0.001 '**' 0.01 '*' 0.05 '.' 0.1 ' ' 1
```

1.1.2.2.: Model summary table

```
## Linear mixed model fit by REML t-tests use Satterthwaite approximations
##   to degrees of freedom [lmerMod]
## Formula: Vividness ~ Condition * Trial.L + (1 | Participant)
##    Data: Data.TargetRealHand.Vividness
##
## REML criterion at convergence: 1767.9
##
## Scaled residuals:
##     Min      1Q  Median      3Q     Max
## -4.6419 -0.6687 -0.0853  0.6596  5.0963
##
## Random effects:
##  Groups      Name        Variance Std.Dev.
##  Participant (Intercept) 4.881    2.209
##  Residual                1.237    1.112
## Number of obs: 537, groups:  Participant, 30
##
## Fixed effects:
##                           Estimate Std. Error        df t value Pr(>|t|)
## (Intercept)                1.71175    0.40906  29.80000   4.185 0.000232
## ConditionRHI sync          1.78021    0.09603 504.00000  18.538  < 2e-16
## Trial.L                    0.61382    0.20469 504.00000   2.999 0.002844
## ConditionRHI sync:Trial.L  0.52897    0.28902 504.00000   1.830 0.067813
##
## (Intercept)               ***
## ConditionRHI sync         ***
## Trial.L                   **
## ConditionRHI sync:Trial.L .
## ---
## Signif. codes:  0 '***' 0.001 '**' 0.01 '*' 0.05 '.' 0.1 ' ' 1
##
## Correlation of Fixed Effects:
##             (Intr) CnRHIs Tril.L
## CndtnRHIsyn -0.118
## Trial.L      0.002 -0.008
## CndRHIs:T.L -0.001  0.009 -0.708
```

1.1.3.: Comparison between the model without an interaction term and model with interaction using likelihood ratio test

```
## refitting model(s) with ML (instead of REML)
```

```
## Data: Data.TargetRealHand.Vividness
## Models:
## object: Vividness ~ Condition + Trial.L + (1 | Participant)
## ..1: Vividness ~ Condition * Trial.L + (1 | Participant)
##        Df    AIC    BIC  logLik deviance  Chisq Chi Df Pr(>Chisq)
## object  5 1775.7 1797.2 -882.87   1765.7
## ..1     6 1774.4 1800.1 -881.19   1762.4 3.3587      1    0.06685 .
## ---
## Signif. codes:  0 '***' 0.001 '**' 0.01 '*' 0.05 '.' 0.1 ' ' 1
```

1.2. "Target: artificial hand" experiment

1.2.1.: Model without interaction between CONDITION and TIME

1.2.1.1.: Anova table

```
## Analysis of Variance Table of type III  with  Satterthwaite
## approximation for degrees of freedom
##           Sum Sq Mean Sq NumDF DenDF F.value    Pr(>F)
## Condition 738.69  738.69     1   501  387.99 < 2.2e-16 ***
## Trial      71.70    8.96     8   501    4.71 1.407e-05 ***
## ---
## Signif. codes:  0 '***' 0.001 '**' 0.01 '*' 0.05 '.' 0.1 ' ' 1
```

1.2.1.2.: Model summary table

```
## Linear mixed model fit by REML t-tests use Satterthwaite approximations
##   to degrees of freedom [lmerMod]
## Formula: Vividness ~ Condition + Trial + (1 | Participant)
##    Data: Data.TargetArtificialHand.Vividness
##
## REML criterion at convergence: 1994.3
##
## Scaled residuals:
##     Min      1Q  Median      3Q     Max
## -2.4066 -0.6771 -0.0691  0.5925  3.7083
##
## Random effects:
##  Groups      Name        Variance Std.Dev.
##  Participant (Intercept) 3.846    1.961
##  Residual                1.904    1.380
## Number of obs: 540, groups:  Participant, 30
##
## Fixed effects:
##                    Estimate Std. Error        df t value Pr(>|t|)
## (Intercept)         1.44415    0.36777  30.60000   3.927 0.000456 ***
## ConditionRHI sync   2.33919    0.11876 501.00000  19.697  < 2e-16 ***
```

```
## Trial.L              1.07006    0.17813 501.00000   6.007 3.64e-09 ***
## Trial.Q              0.04725    0.17813 501.00000   0.265 0.790929
## Trial.C             -0.02351    0.17813 501.00000  -0.132 0.895062
## Trial^4              0.10667    0.17813 501.00000   0.599 0.549549
## Trial^5              0.09770    0.17813 501.00000   0.548 0.583602
## Trial^6              0.10775    0.17813 501.00000   0.605 0.545524
## Trial^7              0.08739    0.17813 501.00000   0.491 0.623952
## Trial^8              0.08329    0.17813 501.00000   0.468 0.640305
## ---
## Signif. codes:  0 '***' 0.001 '**' 0.01 '*' 0.05 '.' 0.1 ' ' 1
##
## Correlation of Fixed Effects:
##             (Intr) CnRHIs Tril.L Tril.Q Tril.C Tril^4 Tril^5 Tril^6 Tril^7
## CndtnRHIsyn -0.161
## Trial.L      0.000  0.000
## Trial.Q      0.000  0.000  0.000
## Trial.C      0.000  0.000  0.000  0.000
## Trial^4      0.000  0.000  0.000  0.000  0.000
## Trial^5      0.000  0.000  0.000  0.000  0.000  0.000
## Trial^6      0.000  0.000  0.000  0.000  0.000  0.000  0.000
## Trial^7      0.000  0.000  0.000  0.000  0.000  0.000  0.000  0.000
## Trial^8      0.000  0.000  0.000  0.000  0.000  0.000  0.000  0.000  0.000
```

1.2.2.: Model with an interaction term between CONDITION and TIME (linear component)

1.2.2.1.: Anova table

```
## Analysis of Variance Table of type III  with  Satterthwaite
## approximation for degrees of freedom
##                  Sum Sq Mean Sq NumDF DenDF F.value    Pr(>F)
## Condition        738.69  738.69     1   507  395.34 < 2.2e-16 ***
## Trial.L           68.70   68.70     1   507   36.77 2.601e-09 ***
## Condition:Trial.L   9.53    9.53     1   507    5.10   0.02438 *
## ---
## Signif. codes:  0 '***' 0.001 '**' 0.01 '*' 0.05 '.' 0.1 ' ' 1
```

1.2.2.2.: Model summary table

```
## Linear mixed model fit by REML t-tests use Satterthwaite approximations
##   to degrees of freedom [lmerMod]
## Formula: Vividness ~ Condition * Trial.L + (1 | Participant)
##    Data: Data.TargetArtificialHand.Vividness
##
## REML criterion at convergence: 1979.7
##
## Scaled residuals:
##     Min      1Q  Median      3Q     Max
## -2.4194 -0.6388 -0.0417  0.5832  3.7486
##
## Random effects:
##  Groups      Name        Variance Std.Dev.
##  Participant (Intercept) 3.848    1.962
##  Residual                1.868    1.367
```

```
## Number of obs: 540, groups:  Participant, 30
##
## Fixed effects:
##                           Estimate Std. Error       df t value Pr(>|t|)
## (Intercept)                 1.4441     0.3677  30.5000   3.928 0.000455
## ConditionRHI sync           2.3392     0.1176 507.0000  19.883  < 2e-16
## Trial.L                     0.6716     0.2496 507.0000   2.691 0.007356
## ConditionRHI sync:Trial.L   0.7969     0.3529 507.0000   2.258 0.024379
##
## (Intercept)               ***
## ConditionRHI sync         ***
## Trial.L                   **
## ConditionRHI sync:Trial.L *
## ---
## Signif. codes:  0 '***' 0.001 '**' 0.01 '*' 0.05 '.' 0.1 ' ' 1
##
## Correlation of Fixed Effects:
##             (Intr) CnRHIs Tril.L
## CndtnRHIsyn -0.160
## Trial.L      0.000  0.000
## CndRHIs:T.L  0.000  0.000 -0.707
```

1.2.3.: Comparison between the model without an interaction term and model with interaction using likelihood ratio test

```
## refitting model(s) with ML (instead of REML)
```

```
## Data: Data.TargetArtificialHand.Vividness
## Models:
## object: Vividness ~ Condition + Trial.L + (1 | Participant)
## ..1: Vividness ~ Condition * Trial.L + (1 | Participant)
##        Df    AIC    BIC  logLik deviance  Chisq Chi Df Pr(>Chisq)
## object  5 1990.3 2011.7 -990.14   1980.3
## ..1     6 1987.2 2012.9 -987.59   1975.2 5.1025      1    0.02389 *
## ---
## Signif. codes:  0 '***' 0.001 '**' 0.01 '*' 0.05 '.' 0.1 ' ' 1
```

2. Models for localization data

2.1. "Target: real hand" experiment

2.1.1.: Model without interaction between CONDITION and TIME

2.1.1.1.: Anova table

```
## Analysis of Variance Table of type III  with  Satterthwaite
## approximation for degrees of freedom
##           Sum Sq Mean Sq NumDF  DenDF F.value    Pr(>F)
## Condition 316.88 158.441     2 752.03 29.8393 3.384e-13 ***
## Trial     250.74  31.342     8 752.02  5.9027 2.398e-07 ***
## ---
## Signif. codes:  0 '***' 0.001 '**' 0.01 '*' 0.05 '.' 0.1 ' ' 1
```

2.1.1.2.: Model summary table

```
## Linear mixed model fit by REML t-tests use Satterthwaite approximations
##   to degrees of freedom [lmerMod]
## Formula: PointingError ~ Condition + Trial + (1 | Participant)
##    Data: Data.TargetRealHand.Localization
##
## REML criterion at convergence: 3703.7
##
## Scaled residuals:
##     Min      1Q  Median      3Q     Max
## -4.5529 -0.6060  0.0108  0.6216  3.0363
##
## Random effects:
##  Groups      Name        Variance Std.Dev.
##  Participant (Intercept) 18.17    4.262
##  Residual                 5.31    2.304
## Number of obs: 792, groups:  Participant, 30
##
## Fixed effects:
##                     Estimate Std. Error        df t value Pr(>|t|)
## (Intercept)         -3.19700    0.79115  30.30000  -4.041 0.000336 ***
## ConditionRHI async  -0.33639    0.20129 752.00000  -1.671 0.095108 .
## ConditionRHI sync   -1.47644    0.20073 752.00000  -7.355 4.99e-13 ***
## Trial.L             -1.55608    0.24656 752.00000  -6.311 4.73e-10 ***
## Trial.Q              0.53912    0.24685 752.00000   2.184 0.029274 *
## Trial.C             -0.18329    0.24616 752.00000  -0.745 0.456748
## Trial^4             -0.20654    0.24555 752.00000  -0.841 0.400540
## Trial^5              0.17156    0.24571 752.00000   0.698 0.485246
## Trial^6              0.08070    0.24457 752.00000   0.330 0.741515
## Trial^7              0.20936    0.24538 752.00000   0.853 0.393815
## Trial^8              0.06737    0.24539 752.00000   0.275 0.783755
## ---
## Signif. codes:  0 '***' 0.001 '**' 0.01 '*' 0.05 '.' 0.1 ' ' 1
##
## Correlation of Fixed Effects:
##             (Intr) CnRHIa CnRHIs Tril.L Tril.Q Tril.C Tril^4 Tril^5 Tril^6
## CndtnRHIasy -0.128
## CndtnRHIsyn -0.128  0.504
## Trial.L      0.001 -0.002 -0.003
## Trial.Q      0.001  0.002 -0.010 -0.003
## Trial.C     -0.001 -0.003  0.002  0.011 -0.001
## Trial^4      0.002 -0.005 -0.006 -0.007  0.007  0.000
## Trial^5      0.000 -0.002  0.005  0.005 -0.008  0.002  0.011
## Trial^6      0.000 -0.003  0.002 -0.005  0.002  0.002  0.004  0.007
## Trial^7      0.000 -0.006 -0.008 -0.002  0.004  0.006  0.000 -0.006  0.002
## Trial^8      0.000 -0.002 -0.003 -0.003  0.006  0.005 -0.003 -0.006  0.000
##             Tril^7
## CndtnRHIasy
## CndtnRHIsyn
## Trial.L
## Trial.Q
## Trial.C
## Trial^4
```

```
## Trial^5
## Trial^6
## Trial^7
## Trial^8      0.009
```

2.1.1.3.: Results from post-hoc comparison of levels of CONDITION

```
##
##   Simultaneous Tests for General Linear Hypotheses
##
## Multiple Comparisons of Means: Tukey Contrasts
##
##
## Fit: lme4::lmer(formula = PointingError ~ Condition + Trial + (1 |
##     Participant), data = Data.TargetRealHand.Localization)
##
## Linear Hypotheses:
##                           Estimate Std. Error z value   Pr(<z)
## RHI async - no hand >= 0   -0.3364     0.2013  -1.671   0.0473 *
## RHI sync - no hand >= 0    -1.4764     0.2007  -7.355 2.86e-13 ***
## RHI sync - RHI async >= 0  -1.1400     0.2001  -5.697 9.15e-09 ***
## ---
## Signif. codes:  0 '***' 0.001 '**' 0.01 '*' 0.05 '.' 0.1 ' ' 1
## (Adjusted p values reported -- fdr method)
```

2.1.2.: Model with an interaction term between CONDITION and TIME (linear and quadratic component)

2.1.2.1.: Anova table

```
## Analysis of Variance Table of type III  with  Satterthwaite
## approximation for degrees of freedom
##                   Sum Sq Mean Sq NumDF  DenDF F.value    Pr(>F)
## Condition         317.51 158.757     2 756.03  30.550 1.743e-13 ***
## Trial.L           211.44 211.440     1 756.03  40.688 3.109e-10 ***
## Trial.Q            25.73  25.731     1 756.02   4.952 0.0263620 *
## Condition:Trial.L  78.64  39.319     2 756.03   7.566 0.0005578 ***
## ---
## Signif. codes:  0 '***' 0.001 '**' 0.01 '*' 0.05 '.' 0.1 ' ' 1
```

2.1.2.2.: Model summary table

```
## Linear mixed model fit by REML t-tests use Satterthwaite approximations
##   to degrees of freedom [lmerMod]
## Formula:
## PointingError ~ Condition + Trial.L + Trial.Q + Condition:Trial.L +
##     (1 | Participant)
##    Data: Data.TargetRealHand.Localization
##
## REML criterion at convergence: 3684.2
##
## Scaled residuals:
```

```
##     Min      1Q  Median      3Q     Max
## -4.5505 -0.6069  0.0137  0.6017  2.8108
##
## Random effects:
##  Groups      Name        Variance Std.Dev.
##  Participant (Intercept) 18.167   4.262
##  Residual                 5.197   2.280
## Number of obs: 792, groups:  Participant, 30
##
## Fixed effects:
##                            Estimate Std. Error       df t value Pr(>|t|)
## (Intercept)                 -3.1935     0.7909  30.3000  -4.038 0.000340
## ConditionRHI async          -0.3412     0.1991 756.0000  -1.713 0.087061
## ConditionRHI sync           -1.4792     0.1986 756.0000  -7.449 2.56e-13
## Trial.L                     -0.5572     0.4263 756.1000  -1.307 0.191564
## Trial.Q                      0.5434     0.2442 756.0000   2.225 0.026362
## ConditionRHI async:Trial.L  -2.2845     0.6017 756.1000  -3.797 0.000158
## ConditionRHI sync:Trial.L   -0.7117     0.5963 756.0000  -1.194 0.233035
##
## (Intercept)                ***
## ConditionRHI async         .
## ConditionRHI sync          ***
## Trial.L
## Trial.Q                    *
## ConditionRHI async:Trial.L ***
## ConditionRHI sync:Trial.L
## ---
## Signif. codes:  0 '***' 0.001 '**' 0.01 '*' 0.05 '.' 0.1 ' ' 1
##
## Correlation of Fixed Effects:
##             (Intr) CnRHIa CnRHIs Tril.L Tril.Q CRHIa:
## CndtnRHIasy -0.127
## CndtnRHIsyn -0.127  0.504
## Trial.L      0.002 -0.007 -0.006
## Trial.Q      0.001  0.002 -0.009  0.000
## CndRHIa:T.L -0.001  0.007  0.004 -0.709 -0.003
## CndRHIs:T.L -0.001  0.005  0.004 -0.715 -0.001  0.507
```

2.1.3.: Comparison between the model without an interaction term and model with interaction using likelihood ratio test

```
## refitting model(s) with ML (instead of REML)
```

```
## Data: Data.TargetRealHand.Localization
## Models:
## object: PointingError ~ Condition + Trial.L + Trial.Q + (1 | Participant)
## ..1: PointingError ~ Condition + Trial.L + Trial.Q + Condition:Trial.L +
## ..1:     (1 | Participant)
##        Df    AIC    BIC  logLik deviance  Chisq Chi Df Pr(>Chisq)
## object  7 3710.9 3743.7 -1848.5   3696.9
## ..1     9 3699.8 3741.9 -1840.9   3681.8 15.103      2  0.0005254 ***
## ---
## Signif. codes:  0 '***' 0.001 '**' 0.01 '*' 0.05 '.' 0.1 ' ' 1
```

2.1. "Target: artificial hand" experiment

2.2.1.: Model without interaction between CONDITION and TIME

2.2.1.1.: Anova table

```
## Analysis of Variance Table of type III  with  Satterthwaite
## approximation for degrees of freedom
##           Sum Sq Mean Sq NumDF  DenDF F.value    Pr(>F)
## Condition 209.58 104.788     2 762.02 19.6225   4.9e-09 ***
## Trial     166.78  20.847     8 762.01  3.9039 0.0001571 ***
## ---
## Signif. codes:  0 '***' 0.001 '**' 0.01 '*' 0.05 '.' 0.1 ' ' 1
```

2.2.1.2.: Model summary table

```
## Linear mixed model fit by REML t-tests use Satterthwaite approximations
##   to degrees of freedom [lmerMod]
## Formula: PointingError ~ Condition + Trial + (1 | Participant)
##    Data: Data.TargetArtificialHand.Localization
##
## REML criterion at convergence: 3741.4
##
## Scaled residuals:
##     Min      1Q  Median      3Q     Max
## -3.4655 -0.6403 -0.0134  0.6361  3.8846
##
## Random effects:
##  Groups      Name        Variance Std.Dev.
##  Participant (Intercept) 11.83    3.440
##  Residual                 5.34    2.311
## Number of obs: 802, groups:  Participant, 30
##
## Fixed effects:
##                     Estimate Std. Error        df t value Pr(>|t|)
## (Intercept)         -3.93825    0.64379  31.00000  -6.117 8.83e-07 ***
## ConditionRHI async   0.47979    0.20030 762.00000   2.395   0.0168 *
## ConditionRHI sync    1.24003    0.19967 762.00000   6.210 8.69e-10 ***
## Trial.L             -1.16155    0.24596 762.00000  -4.723 2.77e-06 ***
## Trial.Q              0.39456    0.24576 762.00000   1.606   0.1088
## Trial.C             -0.18610    0.24555 762.00000  -0.758   0.4488
## Trial^4              0.28006    0.24479 762.00000   1.144   0.2529
## Trial^5             -0.05573    0.24428 762.00000  -0.228   0.8196
## Trial^6             -0.21211    0.24453 762.00000  -0.867   0.3860
## Trial^7             -0.26413    0.24430 762.00000  -1.081   0.2800
## Trial^8             -0.37895    0.24377 762.00000  -1.555   0.1205
## ---
## Signif. codes:  0 '***' 0.001 '**' 0.01 '*' 0.05 '.' 0.1 ' ' 1
##
## Correlation of Fixed Effects:
##             (Intr) CnRHIa CnRHIs Tril.L Tril.Q Tril.C Tril^4 Tril^5 Tril^6
## CndtnRHIasy -0.155
## CndtnRHIsyn -0.156  0.500
```

```
## Trial.L      0.001  0.005 -0.006
## Trial.Q      0.001  0.001 -0.002  0.011
## Trial.C      0.002 -0.005 -0.008  0.010  0.008
## Trial^4      0.001 -0.002  0.000  0.004  0.010  0.006
## Trial^5      0.000 -0.001 -0.002  0.004  0.003  0.004  0.005
## Trial^6      0.001 -0.002 -0.003  0.000  0.001  0.002  0.001  0.003
## Trial^7     -0.001  0.004  0.002  0.003  0.000 -0.003  0.001  0.004  0.001
## Trial^8      0.000  0.000 -0.001  0.000 -0.001  0.000 -0.001  0.000  0.003
##             Tril^7
## CndtnRHIasy
## CndtnRHIsyn
## Trial.L
## Trial.Q
## Trial.C
## Trial^4
## Trial^5
## Trial^6
## Trial^7
## Trial^8      0.000
```

2.1.1.3.: Results from post-hoc comparison of levels of CONDITION

```
##
##   Simultaneous Tests for General Linear Hypotheses
##
## Multiple Comparisons of Means: Tukey Contrasts
##
##
## Fit: lme4::lmer(formula = PointingError ~ Condition + Trial + (1 |
##     Participant), data = Data.TargetArtificialHand.Localization)
##
## Linear Hypotheses:
##                          Estimate Std. Error z value   Pr(>z)
## RHI async - no hand <= 0    0.4798     0.2003   2.395 0.008301 **
## RHI sync - no hand <= 0     1.2400     0.1997   6.210 7.93e-10 ***
## RHI sync - RHI async <= 0   0.7602     0.1999   3.803 0.000107 ***
## ---
## Signif. codes:  0 '***' 0.001 '**' 0.01 '*' 0.05 '.' 0.1 ' ' 1
## (Adjusted p values reported -- fdr method)
```

2.2.2.: Model with an interaction term between CONDITION and TIME (linear component)

2.2.2.1.: Anova table

```
## Analysis of Variance Table of type III  with  Satterthwaite
## approximation for degrees of freedom
##                    Sum Sq Mean Sq NumDF  DenDF F.value    Pr(>F)
## Condition         208.909 104.455     2 767.02 19.5650 5.160e-09 ***
## Trial.L           120.311 120.311     1 767.03 22.5350 2.462e-06 ***
## Condition:Trial.L  21.532  10.766     2 767.04  2.0166    0.1338
## ---
## Signif. codes:  0 '***' 0.001 '**' 0.01 '*' 0.05 '.' 0.1 ' ' 1
```

2.2.2.2.: Model summary table

```
## Linear mixed model fit by REML t-tests use Satterthwaite approximations
##   to degrees of freedom [lmerMod]
## Formula: PointingError ~ Condition * Trial.L + (1 | Participant)
##    Data: Data.TargetArtificialHand.Localization
##
## REML criterion at convergence: 3738
##
## Scaled residuals:
##     Min      1Q  Median      3Q     Max
## -3.2906 -0.6599 -0.0330  0.6637  3.7343
##
## Random effects:
##  Groups      Name        Variance Std.Dev.
##  Participant (Intercept) 11.833   3.440
##  Residual                 5.339   2.311
## Number of obs: 802, groups:  Participant, 30
##
## Fixed effects:
##                           Estimate Std. Error       df t value Pr(>|t|)
## (Intercept)                -3.9382     0.6438  31.0000  -6.117 8.83e-07
## ConditionRHI async          0.4747     0.2003 767.0000   2.370   0.0180
## ConditionRHI sync           1.2374     0.1996 767.0000   6.198 9.33e-10
## Trial.L                    -1.0506     0.4263 767.0000  -2.464   0.0139
## ConditionRHI async:Trial.L  -0.7719     0.6040 767.0000  -1.278   0.2016
## ConditionRHI sync:Trial.L    0.4217     0.6011 767.0000   0.702   0.4832
##
## (Intercept)                ***
## ConditionRHI async         *
## ConditionRHI sync          ***
## Trial.L                    *
## ConditionRHI async:Trial.L
## ConditionRHI sync:Trial.L
## ---
## Signif. codes:  0 '***' 0.001 '**' 0.01 '*' 0.05 '.' 0.1 ' ' 1
##
## Correlation of Fixed Effects:
##             (Intr) CnRHIa CnRHIs Tril.L CRHIa:
## CndtnRHIasy -0.155
## CndtnRHIsyn -0.156  0.500
## Trial.L      0.002 -0.006 -0.006
## CndRHIa:T.L -0.001  0.015  0.005 -0.706
## CndRHIs:T.L -0.001  0.004  0.002 -0.709  0.500
```

2.1.3.: Comparison between the model without an interaction term and model with interaction using likelihood ratio test

```
## refitting model(s) with ML (instead of REML)
```

```
## Data: Data.TargetArtificialHand.Localization
## Models:
## object: PointingError ~ Condition + Trial.L + (1 | Participant)
```

```
## ..1: PointingError ~ Condition * Trial.L + (1 | Participant)
##        Df    AIC    BIC  logLik deviance  Chisq Chi Df Pr(>Chisq)
## object  6 3752.3 3780.4 -1870.2   3740.3
## ..1     8 3752.2 3789.7 -1868.1   3736.2 4.0485      2     0.1321
```
